# Increasing Fruit and Vegetable Consumption Through a Healthy Eating Blog: A Feasibility Study

**DOI:** 10.2196/resprot.6622

**Published:** 2017-04-18

**Authors:** Marie-Eve Caplette, Véronique Provencher, Véronique Bissonnette-Maheux, Marilyn Dugrenier, Annie Lapointe, Marie-Pierre Gagnon, Sharon Straus, Sophie Desroches

**Affiliations:** ^1^ Institute of Nutrition and Functional Foods Laval University Quebec, QC Canada; ^2^ Population Health and Optimal Health Practices Research Unit CHU de Québec Research Center Quebec, QC Canada; ^3^ Faculty of Nursing Laval University Quebec, QC Canada; ^4^ Department of Medicine University of Toronto Toronto, ON Canada

**Keywords:** blogs, nutrition, healthy eating, knowledge translation, feasibility

## Abstract

**Background:**

Despite efforts made by public health organizations to improve consumption of fruits and vegetables, populations in developed countries usually eat less than the minimum recommended. Social media, such as blogs, represent a unique opportunity for improving knowledge translation in health care because they facilitate interactive communication between the public and health professionals. However, no studies have yet evaluated the effect of blogs to promote dietary behavior changes.

**Objective:**

Our study aims to conduct a preliminary assessment before undertaking a full randomized controlled trial (RCT) of the feasibility of using an evidence-based healthy eating blog promoting the consumption of fruits and vegetables among adult women.

**Methods:**

A total of 80 women aged 18 years and older (mean 42, SD 13 years) eating less than five servings per day of fruit and vegetables (mean 2.75, SD 1.84 servings) were recruited. Participants were randomized to the healthy eating blog group (n=40), which included a weekly blog post over a 6-month period, or to a control group (n=40) that had no exposure to the healthy eating blog. Blog posts were written by a registered dietitian and focused on the improvement of fruit and vegetable consumption. We targeted four main determinants of the behavior that were identified as the best predictors for fruit and vegetable intake by two systematic reviews: (1) knowledge, (2) attitude, (3) self-efficacy, and (4) motivation. The intervention was considered feasible if (1) more than 70% of questionnaires were completed, (2) attendance rate was more than 90% for in-person appointments with the research coordinator, (3) participants accessed at least 75% of the blog posts, and (4) the attrition rate was less than 25%. Blog access was assessed by collecting the blog browsing history data for each participant.

**Results:**

During the intervention, 26 posts were published on the blog. Pre- (baseline) and postintervention (6 months) questionnaires were completed by mean 97% (SD 3%) of participants. All participants attended their in-person appointments. Women accessed mean 87% (SD 7%) of the posts published during the intervention. Only 3% (2/80) of participants dropped out of the study. Between the healthy eating blog and control groups, a difference of 1.0 servings of fruits and vegetables (*P*<.001) indicated moderate effects of the intervention (Cohen d=0.54).

**Conclusions:**

These results suggest that an intervention using a healthy eating blog meets preestablished feasibility criteria. A larger-scale RCT using the same methodology will be conducted to assess the impact of a healthy eating blog on the dietary habits of women.

## Introduction

### Healthy Eating

The incidence of chronic diseases has dramatically increased worldwide [1]. The adoption of a healthy diet is recognized as the cornerstone in the prevention and management of chronic diseases [2-4], which are the leading cause of mortality and disability worldwide [1]. One of the best indicators of diet quality is high fruit and vegetable consumption [5], which can help achieve or maintain a healthy body weight [6] and reduce the risk of some cancers [7] and cardiovascular diseases [8]. Accordingly, the World Health Organization recommends a daily consumption of at least 400 grams of fruits and vegetables for the prevention of chronic diseases [9], which corresponds to five servings of fruits and vegetables in Canada’s Food Guide [10]. In Canada, despite the establishment of health promotion initiatives aiming to increase fruit and vegetable consumption, only 40% of Canadians aged 12 years and older consumed at least five servings daily in 2014 [11]. The situation is similar in the United States and in other developed countries [12-14].

Although it is clear that the adoption of preventive behaviors such as a healthy diet is associated with health benefits, nonadherence rates to medication and lifestyle changes are estimated to be 50% in developed countries [15]. Recent systematic reviews have shed light on interventions that best promote dietary behavior change and, thus, adherence to a healthy diet [16]. More specific to fruit and vegetable consumption, systematic reviews aimed at identifying theoretically derived psychosocial determinants of fruit and vegetable intake reported that habit, motivation/goals, beliefs about capabilities/self-efficacy, knowledge, and social support were consistently identified as important determinants of fruit and vegetable consumption [17,18]. Although the previously mentioned systematic reviews contribute to our knowledge about the effective components that should be included in dietary behavior change interventions, much remains unknown about the knowledge translation strategies that should be used to optimally deliver these interventions so that individuals successfully improve their dietary habits.

### Social Media Interventions

Chronic disease prevention and management require sustained lifestyle behavior changes and a long-term commitment, which can require help from a health professional. However, dietary counseling may not be accessible for some patients, such as those living in rural areas, having inflexible working hours and schedule, or having reduced mobility. To overcome these barriers and to inform preventive health care quality improvement, social media interventions in nutrition, such as blogs, could be an effective strategy to reach a large proportion of the Internet population with diverse sociodemographic characteristics, independently of education, race/ethnicity, or health care access [19,20]. Blogs are websites where entries, called *posts*, are written by individuals or a group of individuals including health professionals [21]. Blogs display unique features such as interactivity, social support, and convenience, which could make them valuable additions or alternatives in some cases to traditional face-to-face clinical encounters [22,23]. Another interesting feature of blogs is that bloggers have been found to act as knowledge brokers by playing a crucial role in directing their readers through opinions and hyperlinks [24].

### Purpose of the Feasibility Study

Despite the fact that health blogs are proliferating at high speed, there is no empirically supported knowledge on the impact of health blogs on consumers’ health behaviors and outcomes to promote healthy dietary behavior changes (eg, increase fruit and vegetable intake). To our knowledge, no study has yet evaluated the effects of an evidence-based healthy eating blog on women’s dietary and eating behaviors. Moreover, attrition rates, which refers to the proportion of users who drop out before completion of the study [25], are high in most Web-based health interventions [26-29]. Therefore, as a first step before conducting a randomized controlled trial (RCT), the purpose of this pilot study was to assess the feasibility of a dietary intervention using a healthy eating blog written by a registered dietitian through collecting blog browsing history data for each participant to determine compliance rates, participation rates, and attrition rates.

As a secondary objective, we intended to collect clinical data such as fruit and vegetable consumption and anthropometric measurements. Although this feasibility trial was not powered to detect differences in these outcomes, their assessments will be useful to evaluate the data collection tools. It will also provide an indication of the variance in measurement (effect size) to be used for the power calculation for the definitive RCT.

## Methods

### Study Design

This study was a randomized feasibility trial comparing two groups: control and healthy eating blog (access to the healthy eating blog). The sample size for feasibility trials is typically determined pragmatically, with recommendations of a minimum of 30 participants per group [30,31], which is what we aimed for in our study (n=40 per group to account for dropouts). This study was created according to Thabane’s checklist for pilot studies [32].

### Participants and Recruitment

An advertisement was sent to a list of people who had indicated interest in participating in the Institute of Nutrition and Functional Foods clinical studies. Also, some members of the research team posted the ad on their personal Facebook page. The eligibility of participants was assessed over the phone based on the following inclusion criteria: (1) a woman aged 18 and older living in the Quebec City metropolitan area, (2) has Internet access as well as an active email address, and (3) consumes less than five servings of fruits and vegetables per day (assessed with a 24-hour recall performed by a registered dietitian). We chose to focus the intervention on women because they are primarily responsible for food purchase and preparation in households. Therefore, nutrition promotion strategies that target women have the potential for reach that goes beyond the individual and can thereby improve their family’s health [33,34]. Moreover, a vast majority of dietitian bloggers are women, with a readership predominantly made of women, so our study was shaped around what is available in terms of nutrition blogs [35-37].

Eligible participants were randomized by the research coordinator, who generated a random order list using the Institute of Nutrition and Functional Foods (INAF)’s Web platform. Participants were then scheduled for an in-person individual first visit at the research center to complete a baseline clinical outcomes assessment. All participants gave written informed consent and received Can $100 financial compensation at their final visit at INAF. This project was approved by the ethics committee of Laval University (2014-084 on May 21, 2014).

### Intervention

#### Preliminary Phase

As a preliminary step, in 2013 we performed a qualitative study exploring women’s views and expectations regarding healthy eating blogs as a means to improve their dietary behaviors [38]. Focus groups and individual interviews allowed us to identify the main facilitators and barriers to using a healthy eating blog and preferred key features. The interviews revealed that women preferred blogs that clearly identified the dietitian-blogger: name, picture, academic education, and professional experience. Women also liked beautiful food pictures, recipes that allowed them to apply dietary advice addressed in the posts, and videos showing new cooking techniques. They also mentioned they appreciated when the dietitian-blogger included scientific references at the end of the post. These data were used to develop the healthy eating blog.

#### Intervention Development

The development of the intervention was inspired by the steps of the Intervention Mapping protocol [39] and the results of the preliminary phase described previously [38]. The target behavior of the healthy eating blog was to increase fruit and vegetable consumption. Therefore, blog posts aimed at discussing various positive aspects of healthy eating with a focus on fruit and vegetable intake.

**Figure 1:**
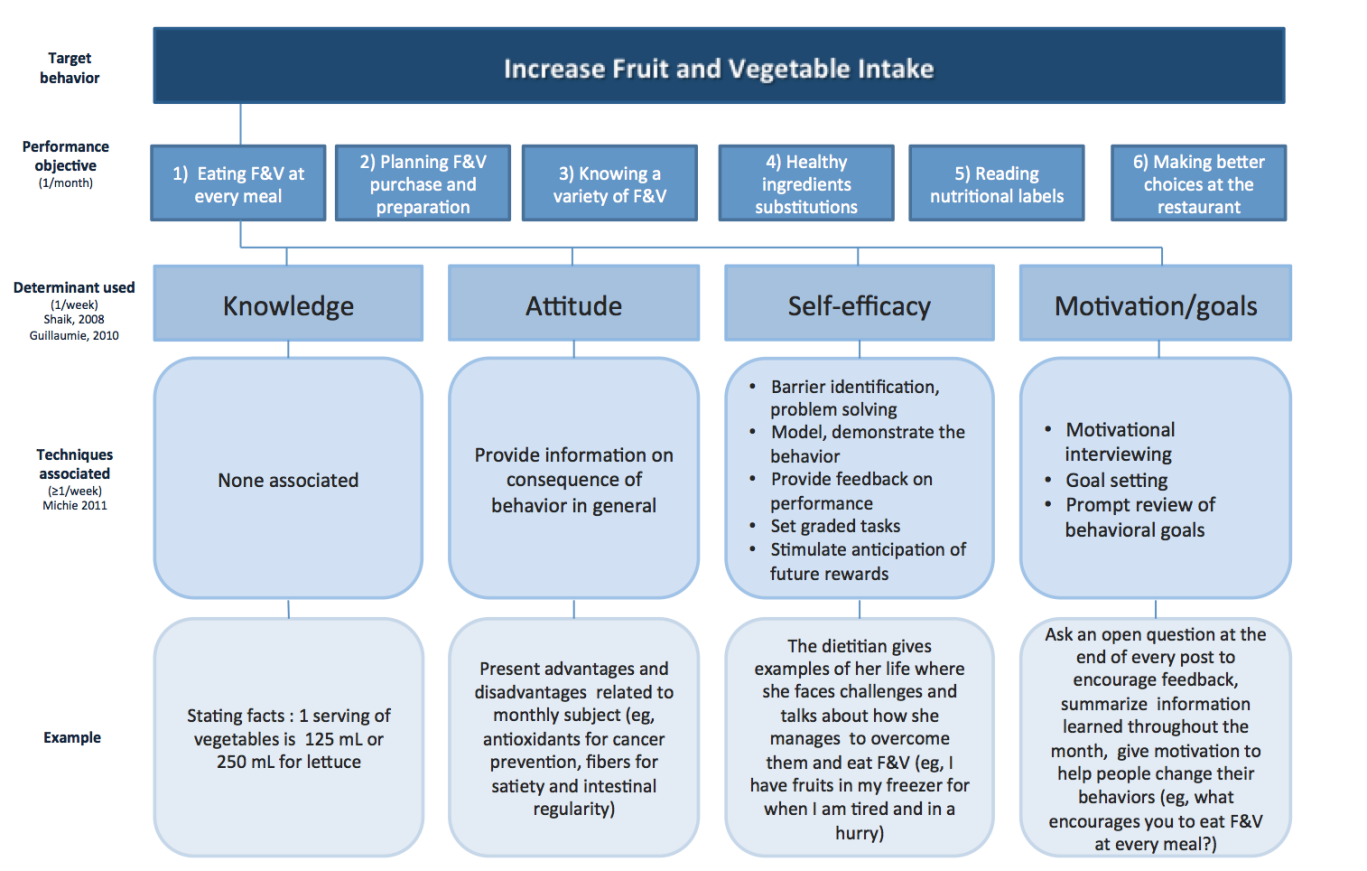
Conceptual framework of the intervention. F&V: fruits and vegetables.

Based on clinical experience of the registered dietitians on the research team and on national and international public health campaigns [40-42], six performance objectives were chosen to improve the target behavior (Figure 1). The performance objectives chosen were, in chronological order of appearance on the blog, (1) eating fruit and vegetables at every meal, (2) planning fruit and vegetables purchase and preparation, (3) knowing a variety of fruit and vegetables, (4) healthy ingredient substitutions in recipes, (5) reading nutritional labels, and (6) making better choices at the restaurant. Based on two recent systematic reviews [17,18] that aimed to review psychosocial determinants of adult fruit and vegetable consumption from different theories and their constructs, we identified the most significant psychosocial determinants of fruit and vegetable consumption applicable to blogs: (1) knowledge, (2) attitude, (3) self-efficacy, and (4) motivation/goals. For each performance objective, we focused on one psychosocial determinant per week. Finally, to promote fruit and vegetable intake through these psychosocial determinants, we used Abraham and Michie’s taxonomy of effective behavior change techniques [43,44]. This taxonomy has been used in multiple studies [45,46] and helped clarify the evidence base about behavior change, allowing specification of interventions in published reports and improving replication, implementation, and evidence synthesis. The platform used to build the blog was WordPress.

#### Intervention Procedure

**Figure 2:**
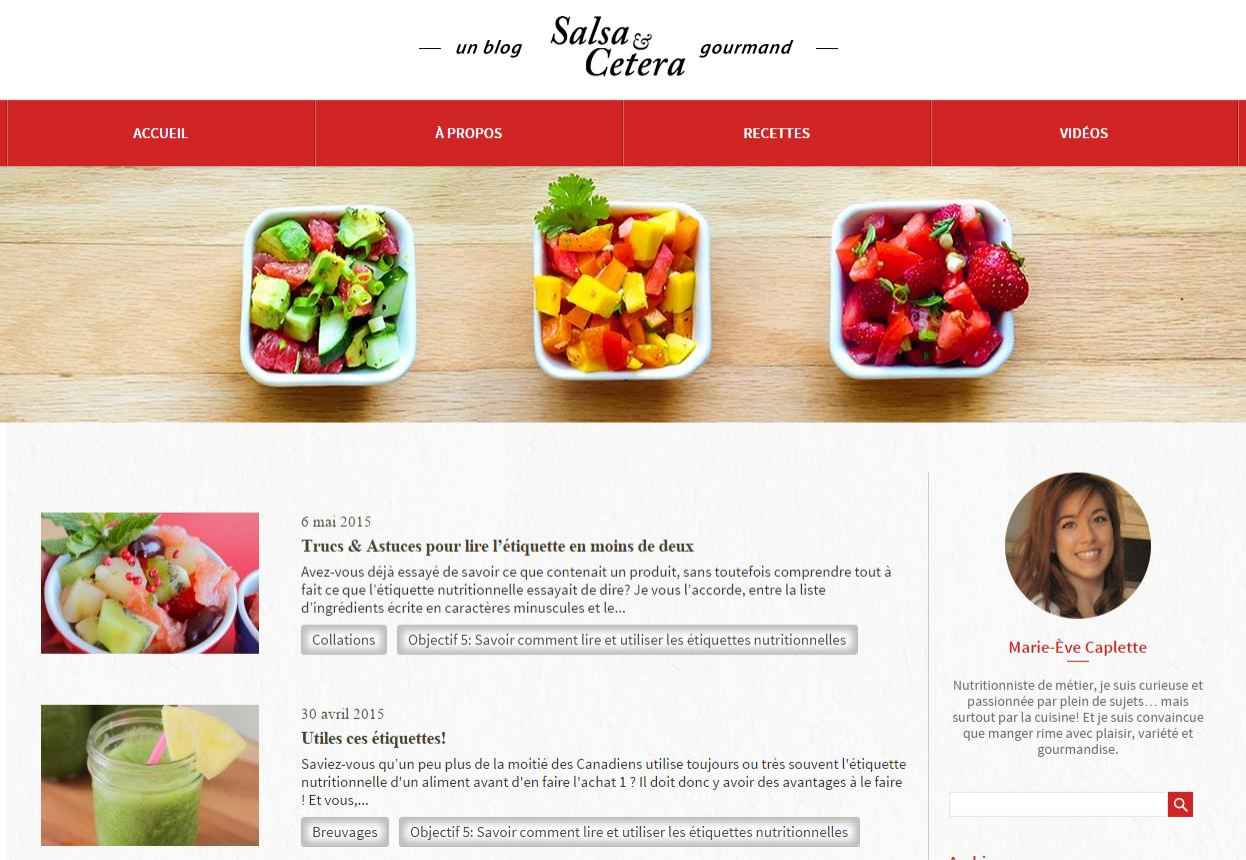
Home page of the intervention healthy eating blog “Salsa Etcetera.”.

The 6-month intervention started on January 13, 2015. Participants received an email to inform them of their allocation (control group or healthy eating blog group). Women in the healthy eating blog group received an identification code and a password to access the healthy eating blog as well as instructions on how to navigate the experimental blog website entitled “Salsa Etcetera” (Figure 2), and how to add comments. They were invited to consult the first two published posts on the healthy eating blog. The research coordinator (coauthor VBM) was available throughout the study to support participants with any issues related to the use of the blog website, while the dietitian-blogger (first author MEC) was primarily responsible for the content of the blog posts, each of which was discussed with coauthors SD and VP. To thank participants in the control group for participating to our study, we gave them a user code and a password after the 6-month intervention that gave them access to the healthy eating blog posts. However, they could not comment or interact with the dietitian-blogger.

Blog posts and comments were read and reviewed by the team of researchers and dietitians before they were published online. Each post included a step-by-step recipe developed by the research team that featured fruits and vegetables. Recipes were described using text and pictures and/or video. Twenty-six posts were created over the 6-month intervention, including the two that were already on the blog when the participants first logged on. Because the majority of women who participated in our preliminary phase mentioned they would like a weekly post to be published (as opposed to twice a week or once every two weeks), participants received a new blog post once a week for 24 weeks. An email was sent every week to inform them that a new post was on the blog. We also sent an email reminder to participants who did not log on the blog for two consecutive weeks.

### Data Collection

All participants were seen at the INAF twice during the study: once at the beginning and once at the end of the 6-month intervention to complete Web-based questionnaires and for anthropometric measurements using standardized procedures (height, weight, and waist circumference) [47]. Body mass index (BMI) was calculated based on measured height and weight. We asked participants to complete a total of six Web questionnaires, including a sociodemographic questionnaire (42 questions), a validated food frequency questionnaire (FFQ; 136 questions) [48], the Three-Factor Eating Questionnaire (a 51-item validated questionnaire) [49], the Restraint Scale (a validated 11-item questionnaire) [50], the Intuitive Eating Scale (a validated 21-item questionnaire) [51], and a social support questionnaire (2 questions). A final questionnaire (4 questions) was added following the intervention to document whether women had been on a specific diet that could affect their food intake over the study period. Participants in the healthy eating blog group also received a monthly Web questionnaire assessing perceived utility of the blog posts. For example, they were asked their opinion about the posts published, if information presented was useful, and if they would use this information in the future.

Feasibility was assessed by evaluating compliance rates, participation rates, and attrition rates. Compliance rates were measured for the 80 participants by the (1) completion of questionnaires assessing clinical outcome data measures for our definitive RCT (change in fruit and vegetable intake, changes in eating behaviors, social support, body weight) and (2) attendance at in-person appointments with the research coordinator. Based on a previous study at INAF that involved the completion of a similar number of questionnaires [52], completion of the questionnaire was considered feasible if it exceeded 70%. The target of attendance for in-person appointments at INAF was 90%. Participation rates were assessed through frequency of use, which was measured primarily by recording the number of posts women accessed out of the 26 published, but also by the number of comments made to the dietitian-blogger’s posts and replies to their peers’ comments initiated by participants. For this specific feasibility outcome, we expected participants to access 75% of the blog posts over the 6 months. The control group did not have access to the blog, so these data were collected for the 40 healthy eating blog participants using Google Analytics and the Web platform (WordPress). Based on a systematic review of Web interventions for changing dietary behaviors [53], we considered the attrition rate acceptable if it was less than 25%.

This feasibility study was not designed to achieve sufficient statistical power to address behavioral outcomes. However, these outcome measures were useful to provide better guidance with regard to development of the definitive RCT. Among the behavioral outcomes of interest, fruit and vegetable intake was assessed with a validated Web-based FFQ [48] at baseline and 6 months. The Web FFQ is an online self-administered quantitative FFQ that allows measuring usual dietary intake over a 1-month period.

### Statistical Analysis

The program SAS was used to analyze data obtained from the questionnaires and to calculate descriptive statistics of the healthy eating blog group and the control group. We used *t* tests to compare the two groups and the generalized linear model procedure to evaluate the effect of time and group on FFQ variables. Means and SD were calculated for the healthy eating blog group. Effect size measures were calculated comparing the mean for fruit and vegetable consumption postintervention of the two groups.

## Results

### Study Recruitment and Baseline Characteristics

**Figure 3:**
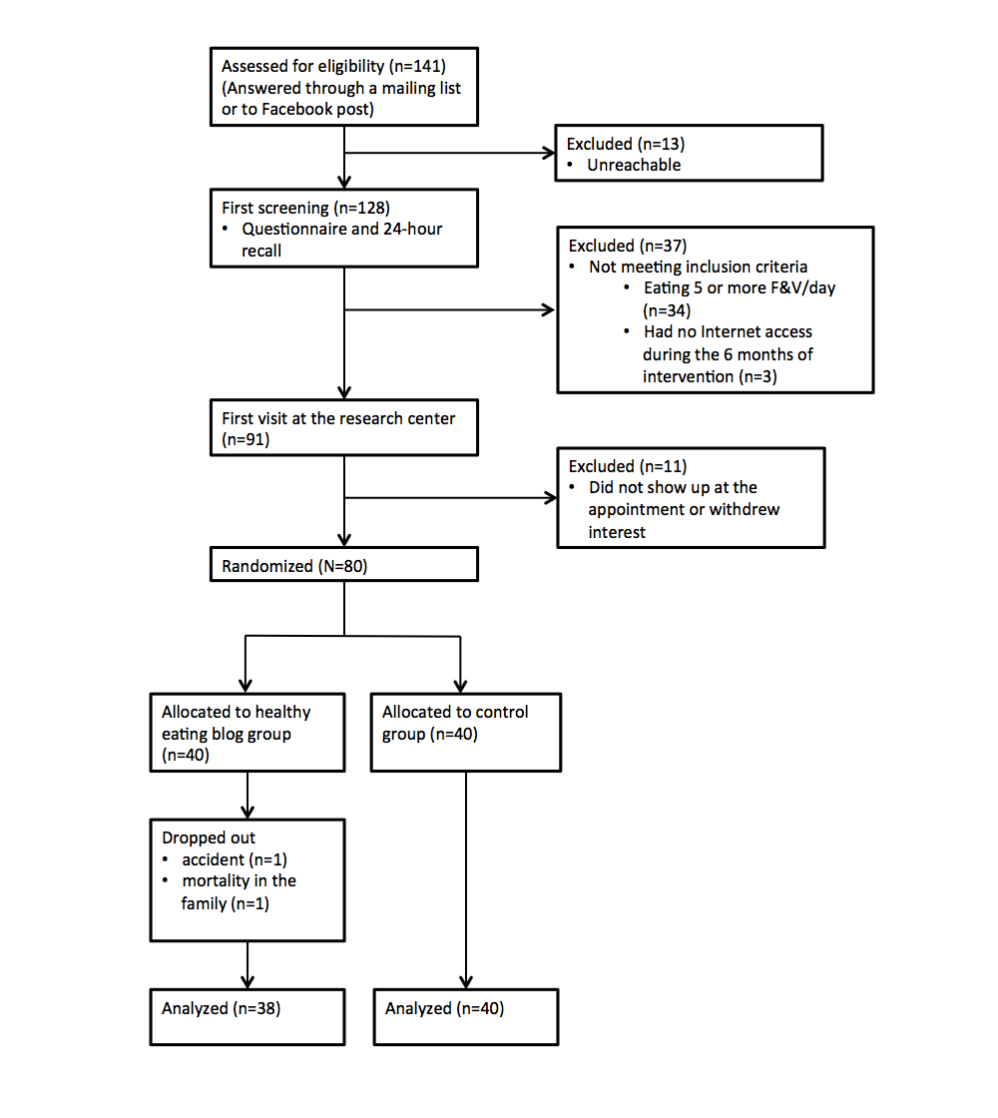
Flowchart of participants. F&V: fruits and vegetables.

Recruitment took place from October 28, 2014 to December 15, 2014 (7 weeks). During this time, eligibility was assessed for 128 women (Figure 3). Among them, 37 were excluded from the first screening for not meeting inclusion criteria (eating ≥5 portions of fruits and vegetables a day: n=34; not having access to Internet during the 6-month intervention: n=3). Eleven eligible participants did not show up at the first appointment and did not express further interest in participating in the study. A total of 80 women (63% of total responding women, n=75 recruited from the mailing list and n=5 recruited on Facebook) enrolled in the study and submitted consent forms at their first visit. Two participants left the study during the first month for personal reasons (one had a concussion and could not use a computer and the second had a death in her family and did not want to participate). Demographic information for participants is described in Table 1.

**Table 1:** Sociodemographic characteristics, eating habits, and Internet use characteristics of participants (N=80).

Sociodemographic, eating habits, and Internet use characteristics		Healthy eating blog (n=40)	Control (n=40)	*P*
Age (years), mean (SD)		42.0 (13.7)	42.2 (13.4)	.95
**Race, n (%)**				>.99
	White	36 (90)	37 (93)	
	Other	4 (10)	3 (8)	
**Education completed, n (%)**				.56
	High school	6 (15)	4 (10)	
	College	15 (38)	13 (33)	
	University	18 (45)	23 (58)	
	Did not answer	1 (2.5)	0 (0)	
**Family income (Can $), n (%)**				.42
	0-19,999	4 (10)	2 (5)	
	20,000-49,999	15 (38)	11 (28)	
	50,000-99,999	12 (30)	12 (30)	
	100,000-149,999	4 (10)	7 (18)	
	150,000-199,999	1 (3)	1 (3)	
	≥200,000	1 (3)	0 (0)	
	Did not answer	3 (8)	7 (18)	
BMI (m/kg^2^), mean (SD)		27.7 (5.2)	27.1 (6.4)	.21
Fruit and vegetable daily intake (servings), mean (SD)		2.45 (1.94)	3.05 (1.70)	.43
**Time spent on Internet for leisure (hours/week), n (%)**				.39
	<1	0 (0)	1 (3)	
	1-2	5 (13)	7 (18)	
	3-4	12 (30)	12(30)	
	5-10	17 (43)	10 (25)	
	≥10	6 (15)	10 (25)	
**Most-used tools for Internet navigation, n (%)**				
	Computer	26 (65)	30 (75)	
	Smartphone	4 (10)	4 (10)	
	Tablet	9 (23)	6 (15)	
	Did not answer	1 (3)	0 (0)	
**Places where Internet is most often used, n (%)**				.85
	Home	34 (85)	36 (90)	
	Work	4 (10)	3 (8)	
	Car or bus	1 (3)	1 (3)	
	Other	1 (3)	0 (0)	
Read a blog before, n (%)		36 (90)	34 (85)	.50
Read a nutrition blog before,^a^ n (%)		22 (55)	20 (50)	.65
Read comments on a blog,^a^ n (%)		29 (73)	27 (68)	.63
Commented on a blog,^a^ n (%)		11 (28)	8 (20)	.43

^a^ Only among participants who had already read a blog before.

Participants were mostly white women aged between 22 and 71 years of age (mean 42, SD 14 years) and ate less than five portions of fruits and vegetables per day (mean 2.75, SD 1.83 servings). The majority of these women received college- or university-level education and more than half of respondents (38/70) had a family income greater than Can $50,000. Mean BMI was 27.5 (SD 4.6), and 31% (25/80) were obese according to Canadian Health Risk Classification (BMI ≥30) [54]. In all, 53% (42/80) had already consulted a blog before. As shown in Table 1, there were no significant differences between the two groups.

### Feasibility Outcomes

#### Compliance Rates

Preintervention and postintervention questionnaires were completed by all participants in both the healthy eating blog and control groups. The monthly questionnaire was completed by 97% (37/38) of participants on average. The lowest completion percentage (92%, 35/38) was observed on month 4. All participants in both groups attended their in-person appointments (100%, 38/38). Therefore, compliance rates all reached our feasibility criteria.

#### Participation Rates

**Figure 4:**
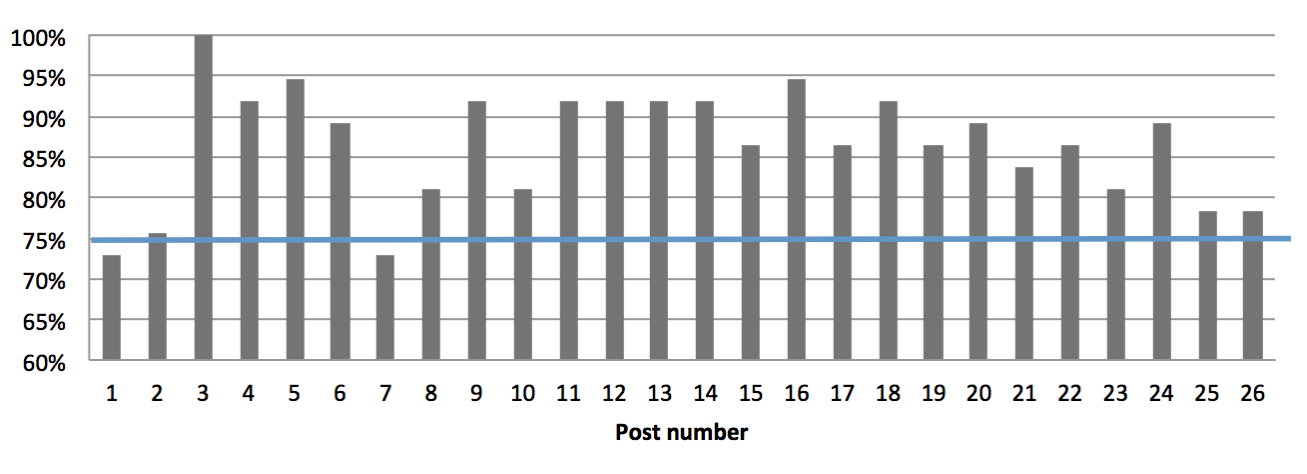
Percentage of participants (n=37) who viewed each post. Note data logs were impossible to collect for one participant who had a firewall installed on her computer. The bold blue line represents the feasibility criterion of 75% of participants viewing each post.

As shown in Figure 4, each weekly post was accessed by at least 73% of women (27/37). We also filmed and edited two videos showing cooking techniques that we posted on YouTube and linked to the blog. Six of 40 participants (16%) viewed the first video, and 4 of 40 participants (11%) viewed the second (Table 2). Participants posted a total of 514 comments on the blog during the intervention. On average, each participant commented 2 (SD 0.4) times per month. Data logs were impossible to collect for one participant who had a firewall installed on her computer. Feasibility criterion was set at 75% of participants viewing each post, as shown by the bold line in Figure 4 **.**

**Table 2:** Participation rates on the healthy eating blog during the 6-month intervention (n=38).^a^

Participation		Month						Total
		1	2	3	4	5	6	
Date range		Jan 13-Feb 11	Feb 12-Mar 11	Mar 12-Apr 8	Apr 9-May 6	May 7-Jun 3	Jun 4-Jul 1	
Articles posted each month, n		6	4	4	4	4	4	26
**Comments**								
	Total comments,^b^ n	141	74	80	73	71	75	514
	Comments/post, mean (SD)	23.5 (9.9)	17.5 (2.0)	16.5 (2.0)	17.0 (5.5)	15.0 (1.5)	16.8 (2.8)	17.7 (4.7)
	Comments/participant,^b^ mean (SD)	3.7 (0.3)	1.9 (0.2)	2.1 (0.1)	1.9 (0)	1.9 (0.1)	2.0 (0.0)	13.5 (0.7)
**Posts, recipes, and videos, n**								
	Total printed recipes^b,c^	35	19	6	10	30	27	127
	Total video views^b,c,d^	0	0	0	0	6	4	10

^a^ n=38 because two participants dropped out.

^b^ For the month’s new posts and precedent posts.

^c^ n=37 because data logs were impossible to collect for one participant who had a firewall installed on her computer.

^d^ First video was posted on May 6 and the second video was posted on June 4. All four views during month 6 were for video 2.

#### Attrition Rate

The attrition rate was 3% (2/80), which is less than our preestablished 25% criteria. The two participants who dropped out were in the healthy eating blog group and the reasons were external to the study as mentioned previously.

#### Clinical Outcomes

**Figure 5:**
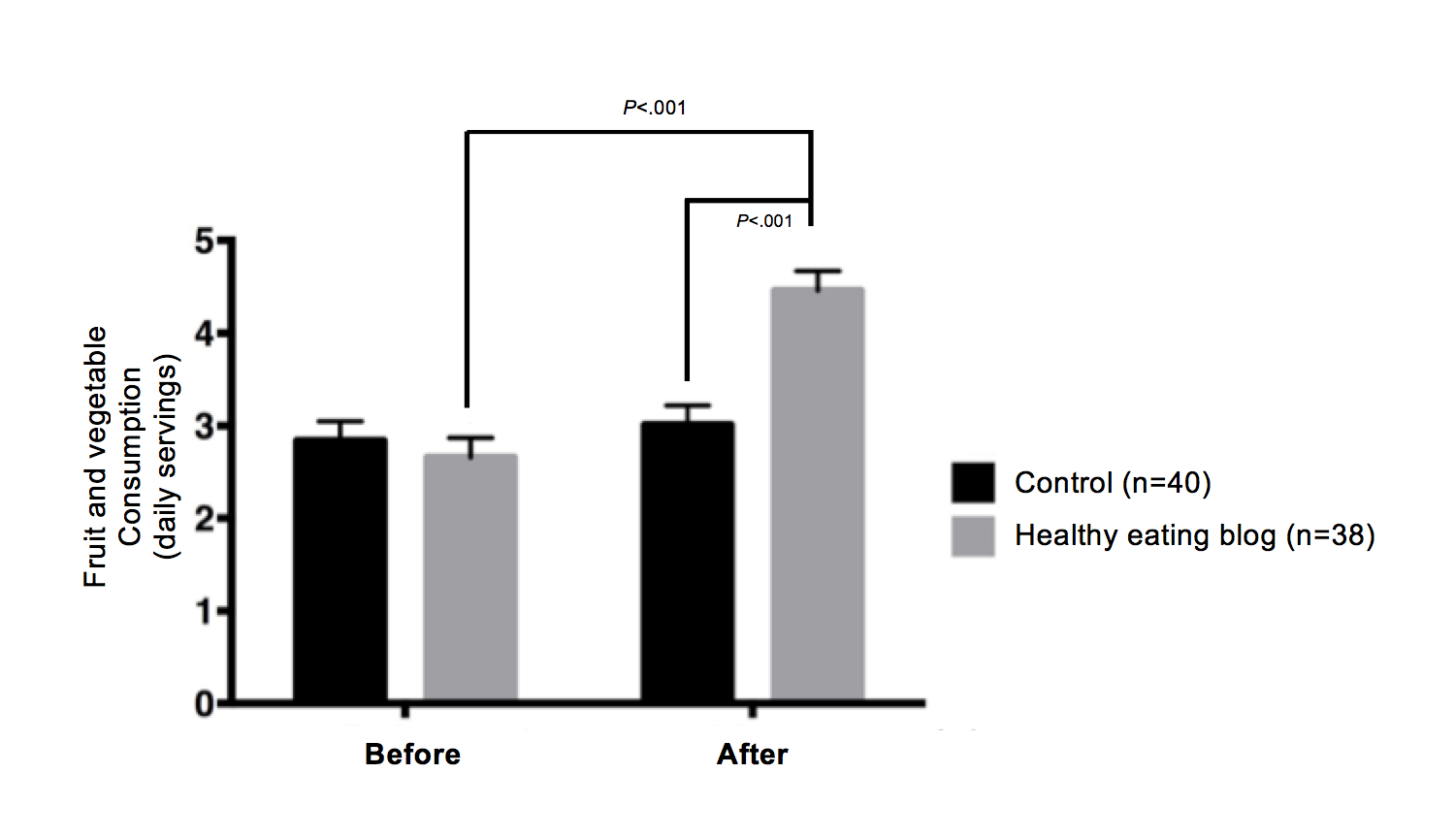
Mean fruit and vegetable consumption for the two groups before and after the 6-month intervention (n=78).

We observed no significant differences in fruit and vegetable consumption between the healthy eating blog group (mean 2.44, SD 1.91 portions/day) and the control group (mean 3.05, SD 1.70 portions/day) at baseline (Figure 5). However, the healthy eating blog group significantly increased their fruit and vegetable consumption at the 6-month visit (mean 4.23, SD 1.85 portions/day, *P*<.001; mean difference 1.79, SD 2.47 portions/day), and this was significantly different from the control group (mean 3.22, SD 1.86 portions/day, *P*<.001). No significant changes in other food groups were found. The difference of 1.0 servings of fruits and vegetables (*P*<.001) found between groups indicated moderate effects of the intervention (Cohen *d*=0.54). There were no significant differences between anthropometric measurements before and after the intervention (Cohen *d*=0.14).

Figure 5. Mean fruit and vegetable consumption for the two groups before and after the 6-month intervention (n=78).

## Discussion

Our study is the first to evaluate the feasibility of an intervention using an evidence-based healthy eating blog to improve women’s dietary and eating behaviors. According to our preestablished criteria, the intervention is deemed feasible.

### Compliance Rates

Measured completion of pre- and postintervention questionnaires (100%) and in-person appointment attendance (100%) met our preestablished compliance rate criteria. Unsurprisingly, all questionnaires were completed because participants filled them in during their in-person appointment, which everyone attended. Compliance rates were high for both the healthy eating blog and control groups. The incentive of Can $100 given at the end of the study may have contributed to compliance. Completion of the monthly questionnaires by the healthy eating blog group (mean 96.5%, SD 3.2%) also met our compliance rate criteria of 70%. The reminder sent to participants when they did not complete the monthly questionnaire may have contributed to achieving this high completion rate.

### Participation Rates

It is known that increased participant use of and engagement with an intervention platform are associated with greater behavior change [55]. Our criterion to assess participation was for participants to view at least 75% of published blog posts. This criterion was met; participants viewed a mean 86.6% of posts. Only two blog posts were seen by less than 75% of participants (73% for posts 1 and 7).

In concordance with studies included in Williams et al’s systematic review of RCTs examining the use of social media to promote healthy diet and exercise in the general population [26], participation rates, measured by the number of pages viewed and the number of comments, were higher in the first month. Higher participation in our study could probably be explained by the fact that two blog posts were published before the study onset so that participants would enter the study and find an active blog. The challenge, as identified by Williams et al [26], is to maintain adherence and keep the participants engaged. However, unlike studies included in this review, we did not find a decrease in usage throughout the intervention period. This could have been due to selection of the intervention components that led to a sustained interest from participants. The fact that we responded to all comments posted on the blog by participants in a timely fashion might have helped to optimize participation.

Participants in our preliminary study [38], and in other studies as well [27,56], mentioned that receiving an email when a new post was published was a useful reminder to engage with the blog. A review examining mobile health interventions for lifestyle behavior changes mentioned that reminders can improve adherence to behavioral goals [57]. This, along with reminders sent to participants who did not log on to the blog for two consecutive weeks, could have helped maintain adherence to the intervention.

We did not have a criterion for the number of comments on the blog, but compared to existing blogs, the participation rate was very high [58]. The fact that comments were posted anonymously (participants were named Blog-pilot X) could have encouraged participants to publish comments without apprehensions regarding privacy [59]. Using a narrative approach and asking a question to blog readers at the end of all posts could have positively influenced interaction [38,60].

Only a few people watched published videos (16% for video 1 and 11% for video 2). This is consistent with findings from Strekalova et al [61], who concluded that messages with photos are more effective than videos for encouraging public engagement on Facebook. In our preliminary study [38], 45% (15/33) of women mentioned that videos could be useful; however, 27% (9/33) thought they were not very relevant and 30% (10/33) mentioned not having enough time to watch them. In a RCT comparing a video and text version of a Web-based computer-tailored intervention for obesity prevention, the video version was the most effective intervention and most appreciated [62]. Therefore, videos could prove useful for reaching a certain portion of the population, for instance less-educated people who could have a lower literacy level [63], so blog interventions could be adapted according to the target audience. Because videos were not popular in this pilot study, we will not include them in the future RCT.

### Attrition Rate

Attrition rate was very low (3%, 2/80). This could be due to the honorarium given to participants in both the control group and healthy eating blog group at the end of the study (Can $100). We also think that this study did not imply much time and involvement because participants could log on to the blog from home and were not forced to write comments and interact with the dietitian-blogger or other participants. A recent study examined the feasibility of delivering a group Web-based and face-to-face weight-loss intervention to 40 young adults with low income and reported that 30% of participants completed the 5-month intervention [56]. This attrition is high, and higher numbers have been observed in other Web-based weight-loss interventions [64,65]. A systematic review of 12 computer-tailored dietary behavior change interventions identified some commonalities between the five studies with higher retention rates: “all used highly motivated and/or self-select samples; a majority were intended as multiple exposure interventions ranging from two to six months and a majority offered incentives to participants” [53].

### Limitations of the Study

Our sample may not be representative of the general population. According to a 2014 report by the Pew Research Center, users of social media in the United States are predominantly aged between 18 and 34 years [66]. Also, a cross-sectional survey on health-related communication trends and practices concluded that a significant linear relationship was observed between younger age and blogging site participation [19]. Our sample had a mean age of 42.0 (SD 13.7) years, which is older than what is observed for users of social media. Posting an ad on the personal Facebook page of the research team members may have created a bias due to the demographics of the research team; a more active Facebook or Twitter recruitment could have helped to recruit younger people [67]. Moreover, participants in our study were mostly white, well educated (college or more), and had a relatively high family income. According to a survey conducted by the Pew Research Center in 2014, the majority of adults who are active on social media platforms have a family income less than US $30,000 [68]. In order to reach a more diverse audience, other recruitment methods for the RCT, such as posting an ad in local newspapers and in community centers, should be considered.

Finally, we faced some difficulties in obtaining records of log-ins. Depending on the WordPress updates (the Web software used to create the blog), we noticed that statistics were erroneous for a small number of participants whose log-in statistics were not recorded for some posts, although they had commented on these same posts. This is why we chose to focus on the number of comments and the number of page views to assess participation rates, and not the number of log-ins, which was a less reliable statistic.

### Conclusion

This feasibility study provided valuable information about how to optimize and implement a healthy eating blog intervention. It also contributed to inform the conduct of a future definitive RCT to assess the efficacy of a healthy eating blog to improve dietary and eating behaviors. Because preestablished feasibility criteria were met, characteristics of the intervention to be used in the RCT will remain unchanged. Given the novelty of this intervention, this project paves the way for designing and evaluating the effects of other social media tools on consumers’ health outcomes. This study was the first to provide an empirically supported basis for the design of interventions using social media, more specifically blogs, to improve the quality of health promotion and disease prevention health care services through enhanced bidirectional exchange of evidence-based and experiential nutrition-related knowledge.

To date, there is very modest evidence that interventions using online social media may be effective. Because the Web is used more and more and this field of research is very recent [29], more research is needed to determine the effects of a blog on dietary and eating behaviors.

## Abbreviations

BMI: body mass index
FFQ: food frequency questionnaire
INAF: Institute of Nutrition and Functional Foods
RCT: randomized controlled trial
